# Role of ACSL4 in modulating farnesoid X receptor expression and M2 macrophage polarization in HBV‐induced hepatocellular carcinoma

**DOI:** 10.1002/mco2.706

**Published:** 2024-09-12

**Authors:** Wenbiao Chen, Huixuan Xu, Liliangzi Guo, Fengping Zheng, Jun Yao, Lisheng Wang

**Affiliations:** ^1^ Department of Gastroenterology Shenzhen People's Hospital The Second Clinical Medical College Jinan University The First Affiliated Hospital Southern University of Science and Technology Shenzhen China; ^2^ Department of Rheumatology and Immunology The Second Clinical Medical College Jinan University (Shenzhen People's Hospital) Shenzhen China; ^3^ Shenzhen Peking University‐The Hong Kong University of Science and Technology Medical Center Peking University Shenzhen Hospital Shenzhen Guangdong China

**Keywords:** ACSL4, bile acid metabolism, farnesoid X receptor, hepatitis B virus‐hepatocellular carcinoma, M2 macrophage polarization

## Abstract

The intricate relationship between bile acid (BA) metabolism, M2 macrophage polarization, and hepatitis B virus‐hepatocellular carcinoma (HBV‐HCC) necessitates a thorough investigation of ACSL4's (acyl‐CoA synthetase long‐chain family member 4) role. This study combines advanced bioinformatics and experimental methods to elucidate ACSL4's significance in HBV‐HCC development. Using bioinformatics, we identified differentially expressed genes in HBV‐HCC. STRING and gene set enrichment analysis analyses were employed to pinpoint critical genes and pathways. Immunoinfiltration analysis, along with in vitro and in vivo experiments, assessed M2 macrophage polarization and related factors. ACSL4 emerged as a pivotal gene influencing HBV‐HCC. In HBV‐HCC liver tissues, ACSL4 exhibited upregulation, along with increased levels of M2 macrophage markers and BA. Silencing ACSL4 led to heightened farnesoid X receptor (FXR) expression, reduced BA levels, and hindered M2 macrophage polarization, thereby improving HBV‐HCC conditions. This study underscores ACSL4's significant role in HBV‐HCC progression. ACSL4 modulates BA‐mediated M2 macrophage polarization and FXR expression, shedding light on potential therapeutic targets and novel insights into HBV‐HCC pathogenesis.

## Graphical abstract

Molecular mechanisms of ACSL4‐mediated bile acid metabolism and nuclear receptor‐mediated M2 macrophage polarization in HBV‐HCC development.

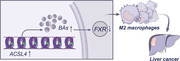

## 1 INTRODUCTION

The infection of hepatitis B virus (HBV) poses a significant worldwide public health challenge, as certain cases may progress to HBV‐related hepatocellular carcinoma (HBV‐HCC).^1^, ^2^, ^3^, ^4^ Hepatocellular carcinoma (HCC) is a prevalent malignancy associated with a grave prognosis, especially in Asia.^5^, ^6^, ^7^, ^8^ Although the promotion of the HBV vaccine has reduced the number of new infections, the incidence of HBV‐HCC continues to rise annually.^9^ Understanding the pathological mechanisms of HBV‐HCC is a challenging task due to the complexity of HBV infection and its intricate interactions with liver cancer.^2^ The elevated occurrence and fatality rates of HBV‐related liver cancer in clinical settings^10^ make it crucial to study the disease progression and adverse prognosis in HBV‐HCC patients for effective HCC treatment.

Research has shown that the involvement of M2 macrophages is pivotal in shaping the tumor microenvironment, with a significant focus on the initiation and advancement of liver malignancy.^11^, ^12^, ^13^, ^14^, ^15^ Signals released by tumor cells and other immune cells can polarize macrophages into the M2 subtype, forming tumor‐associated macrophages. These M2 macrophages have been found to promote tumor growth and metastasis.^14^, ^16^, ^17^

Bile acids (BAs), as essential digestive components secreted by the liver, also have notable implications in the pathogenesis of liver conditions and liver malignancies.^18^, ^19^, ^20^ Research has shown that in pathological liver conditions, there are abnormal changes in the total BA levels in the body. Some of these BA alterations can impact the activity of specific BA receptors, subsequently influencing the occurrence and progression of HCC through mechanisms such as immune inflammation or cell apoptosis. For instance, the farnesoid X receptor (FXR), G protein‐coupled receptors 1, pregnane X receptor, constitutive androstane receptor, and sphingosine‐1‐phosphate receptor 2 have all been confirmed to affect the development of HCC through various pathways.^21^ Recent studies have further elucidated the connection between BA metabolism and M2 macrophage polarization, although the underlying molecular mechanisms remain incompletely understood.^22^

The enzyme acyl‐CoA synthetase long‐chain family member 4 (ACSL4), associated with fatty acid metabolism, has emerged as a key player in the progression of liver cancer in recent studies.^23^, ^24^ ACSL4 regulates various biological processes, including fatty acid oxidation and synthesis, thereby influencing cell proliferation, migration, and invasion.^25^, ^26^, ^27^ Simultaneously, the nuclear receptor FXR is also considered a crucial factor in regulating BA metabolism.^28^, ^29^, ^30^, ^31^ However, the mechanism by which ACSL4 regulates BA metabolism through FXR and further impacts M2 macrophage polarization and liver cancer progression remains unclear.

To gain a deeper understanding of how ACSL4 regulates BA metabolism and its impact on HBV‐HCC, this study plans to explore its mechanisms using bioinformatics analysis, in vitro cell experiments, and in vivo mouse models. The research aims to uncover the interactions between ACSL4, BAs, FXR, and M2 macrophage polarization, and how these factors collectively influence the occurrence and development of HBV‐HCC. The results of this study may provide fresh tactics and goals for thwarting and managing HBV‐HCC.

In summary, by delving into the interaction between ACSL4 and BA metabolism, and how these interactions affect the occurrence and development of HBV‐HCC through FXR and macrophage polarization, this study will offer fresh perspectives into the intricate molecular mechanisms underlying liver cancer and may guide future therapeutic approaches for this disease.

## 2 RESULTS

### 2.1 Multichip joint analysis was conducted to screen critical genes involved in HBV‐HCC

**FIGURE 1:**
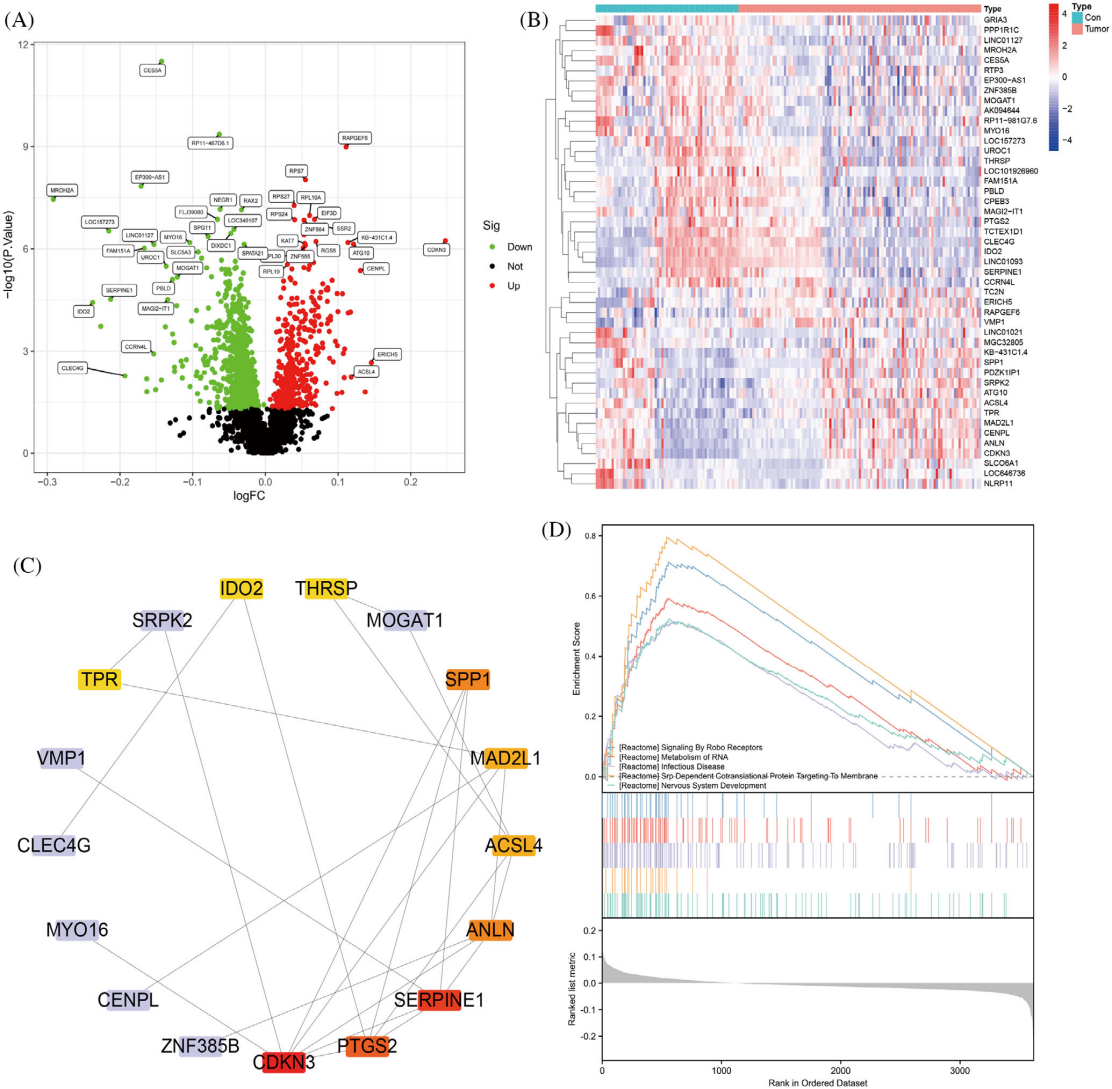
Identification of essential genes related to HBV‐HCC through bioinformatics analysis. (A and B) Heatmap and volcano plot of the merged datasets GSE55092 and GSE121248 comparing normal control samples (normal group, *n* = 63) with liver cancer tissue samples (HBV‐HCC group, *n* = 107). (C) PPI analysis of differentially expressed genes from the combined analysis of GSE55092 and GSE121248 datasets, where red indicates the highest degree value and yellow signifies lower degree values. (D) Pathway enrichment analysis of GSEA from the combined analysis of GSE55092 and GSE121248 datasets. HBV‐HCC, hepatitis B virus‐hepatocellular carcinoma; GSEA, gene set enrichment analysis; PPI, protein–protein interaction.

To identify key genetic factors related to HBV‐HCC, we accessed the chip datasets GSE55092 and GSE121248 from the Gene Expression Omnibus (GEO) database. After merging and analyzing the datasets, a total of 4840 genes were obtained. Filtering was carried out using a threshold of *p* < 0.05, resulting in the selection of 47 differentially expressed genes. Subsequently, volcano plots and heatmaps were generated to visualize the findings (Figure 1A,B). The proteins encoded by the 47 genes that showed differential expression underwent analysis for protein–protein interactions (PPI), resulting in the identification of 17 highly correlated key genes. Utilizing the cytoHubba plugin in Cytoscape, these genes were ranked based on their degree values in descending order. The top 10 genes included CDKN3, ANLN, PTGS2, SERPINE1, ACSL4, MAD2L1, SPP1, IDO2, THRSP, and TPR, with degree values of 7, 5, 5, 5, 4, 4, 3, 2, 2, and 2, respectively (Figure 1C). These genes have a pivotal function in the progression of hepatic fibrosis and HBV‐HCC.

Gene set enrichment analysis (GSEA) revealed pathways that exhibited significant associations with the specified 10 genes, including SIGNALING_BY_ROBO_RECEPTORS, METABOLISM_OF_RNA, INFECTIOUS_DISEASE, SRP_DEPENDENT_COTRANSLATIONAL_PROTEIN_TARGETING_TO_MEMBRANE, and NERVOUS_SYSTEM_DEVELOPMENT (Figure 1D). Further literature review revealed a close correlation between HBV‐HCC and the METABOLISM_OF_RNA pathway.^32^, ^33^

### 2.2 Analysis of immune infiltration reveals the correlation between M2 macrophages and HBV‐HCC

**FIGURE 2:**
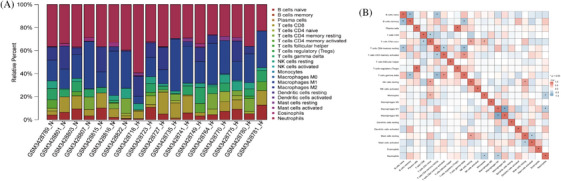
Immune infiltration analysis of HBV‐HCC and M2 macrophages. (A) Immune infiltration plot comparing standard control samples (normal group, *n* = 7) with hepatocellular carcinoma samples (HBV‐HCC group, *n* = 11) in dataset GSE121248. The *x*‐axis represents the differences in immune cell populations between samples, and the height of the bars represents the expression level of immune infiltration in different samples. Higher bars indicate higher levels of immune cells or immune‐related gene expression, while lower bars indicate lower levels of immune infiltration. (B) Correlation analysis heatmap of immune cells, where *p < 0.05, red represents positive correlation and blue represents negative correlation. HBV‐HCC, hepatitis B virus‐hepatocellular carcinoma.

To explore the molecular basis for the influence of M2 polarization of macrophages on HBV‐HCC, we conducted an immune infiltration analysis of the HBV‐HCC dataset in the GEO database and analyzed the correlation between critical genes and M2 macrophages. Immunoinfiltration analysis results show that in HBV‐HCC samples, there is a higher relative abundance of M2 macrophages (Figure 2A); M2 macrophages have a strong correlation with M1 macrophages (Figure 2B).

The above results indicate that immunoinfiltration analysis reveals a specific correlation between M2 macrophages and the occurrence and development of HBV‐HCC.

### 2.3 Clinical experiments verify that ACSL4 regulates BA metabolism and FXR influences M2 macrophage polarization

In a multichip integrated analysis aimed at identifying key genes and pathway factors involved in HBV‐HCC development, we conducted a thorough literature search. Our findings revealed that ACSL4,^34^, ^35^ CDKN3,^36^, ^37^ SERPINE1,^38^, ^39^ PTGS2,^40^ ANLN,^41^, ^42^ SPP1,^43^ TPR,^44^ MAD2L1,^45^ IDO2,^46^ and THRSP^47^ are implicated in metabolism. Subsequently, we gathered samples of cancerous tissue from HBV‐HCC patients along with paired adjacent normal tissue for analysis.

**FIGURE 3:**
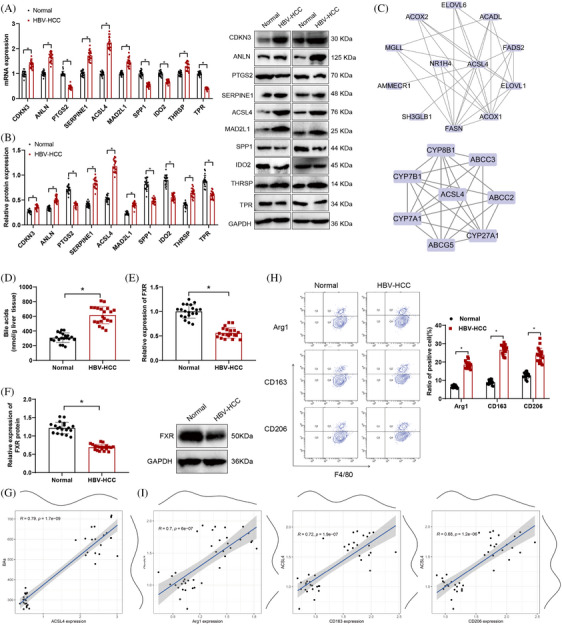
Levels of ACSL4, BAs, FXR, and M2 macrophage polarization markers in normal and HCC tissues. (A and B) mRNA and protein levels of critical genes in adjacent normal tissue (normal group) and cancer tissue (HBV‐HCC group) of HBV‐HCC patients, detected by RT‐qPCR and Western blot respectively, *n* = 20, indicating statistical significance between the two groups at *p* < 0.05. (C) Protein interaction analysis of ACSL4 with BA‐related proteins encoded by genes such as CYP7A1, CYP27A1, CYP8B1, and with FXR, where the outer circle represents genes associated with ACSL4, and NR1H4 is the scientific name for FXR. (D) Total BA levels detected by mass spectrometry in adjacent normal tissue (normal group) and cancer tissue (HBV‐HCC group), *n* = 20, indicating statistical significance between the two groups at *p* < 0.05. (E) Expression levels of FXR detected by RT‐qPCR in adjacent normal tissue (normal group) and cancer tissue (HBV‐HCC group), *n* = 20, indicating statistical significance between the two groups at *p* < 0.05. (F) WB to detect the protein expression levels of FXR in adjacent normal tissues (normal group) and cancer tissues (HBV‐HCC group), with *n* = 20, demonstrating a significant difference between the two groups, *p* < 0.05. (G) The correlation analysis between the mRNA levels of the key gene ACSL4 and BA levels, where *p* < 0.05 indicates a statistical difference. *R* denotes the correlation coefficient, where *R* > 0 signifies a positive correlation and *R* < 0 indicates a negative correlation. The closer the value is to 0, the weaker the correlation. The external curve represents the marginal histogram of a single variable. (H) Flow cytometry to measure the expression levels of the M2 macrophage polarization markers Arg1, CD163, and CD206, showing a significant difference between the two groups with *p* < 0.05. (I) Represents the correlation analysis between the mRNA levels of ACSL4 and the expression levels of the M2 macrophage polarization markers Arg1, CD163, and CD206, where *p* < 0.05 indicates a statistical difference. *R* denotes the correlation coefficient, and the external curve represents the marginal histogram of a single variable. HBV‐HCC, hepatitis B virus‐hepatocellular carcinoma; FXR, farnesoid X receptor.

Examination through RT‐qPCR and Western blot assays revealed that in HBV‐HCC tissues, the expression levels of ACSL4, CDKN3, SERPINE1, ANLN, MAD2L1, and THRSP were upregulated, while the expression levels of PTGS2, SPP1, TPR, and IDO2 were downregulated. Among these 10 genes, the expression level of ACSL4 is higher than other genes (Figure 3A,B). After reviewing the literature, it was found that the development and evolution of HBV‐HCC are intricately linked to ACSL4 and BAs.^34^, ^35^, ^48^ We also performed survival analysis applying the GEPIA database (http://gepia.cancer‐pku.cn/index.html). Within TCGA‐LIHC (liver HCC) dataset, both the *p* value from the Logrank test and the significance test *p* value for HR indicated that the expression level of ACSL4 significantly impacts patient survival rates. High ACSL4 expression was significantly associated with poorer overall survival. Therefore, ACSL4 may serve as a potential prognostic marker guiding clinical treatment strategies (Figure S1J).

Therefore, we conducted PPI analysis of ACSL4 with proteins encoded by BA genes and FXR, revealing that ACSL4 interacts with BAs and FXR, suggesting a regulatory relationship (Figure 3C). Mass spectrometry results indicated elevated BA levels in HBV‐HCC compared with normal tissues, while FXR mRNA and protein expression was downregulated as demonstrated by RT‐qPCR and Western blot analysis in HBV‐HCC compared with normal tissues (Figure 3D,F). Furthermore, correlation analysis between key genes, pathway factors, and BAs demonstrated a positive correlation between ACSL4 and BA levels (Figures 3G and S1).

The flow cytometry findings indicated increased expression of Arg1, CD163, and CD206 in HBV‐HCC tissues in contrast to normal tissue samples (Figure 3H), suggesting the presence of polarized M2 macrophages in HBV‐HCC. In addition, an investigation was performed to assess the correlation between ACSL4 mRNA expression levels and the markers indicating polarization toward M2 macrophages. A robust positive correlation was observed between ACSL4 expression levels and the expression levels of Arg1, CD163, and CD206 in the study results (Figure 3I). The above results indicate that ACSL4 may affect the polarization of M2 macrophages and lead to the occurrence of HBV‐HCC by supervising the BAs metabolism and influencing the FXR expression.

### 2.4 ACSL4 regulates BAs and FXR to influence the occurrence and progression of HCC cells

To authenticate the regulatory function of ACSL4 in the initiation and progression of HCC through BAs and the FXR, we conducted experiments using human hepatic stellate cells (LX‐2) to measure the levels of ACSL4, BAs, and FXR, as well as their effects on the proliferation, migration, and invasive potential of HCC cells. Examination was conducted on the presence of ACSL4 and FXR in HBV‐infected hepatic stellate cells (HBV‐LX‐2) using Western blot.

**FIGURE 4:**
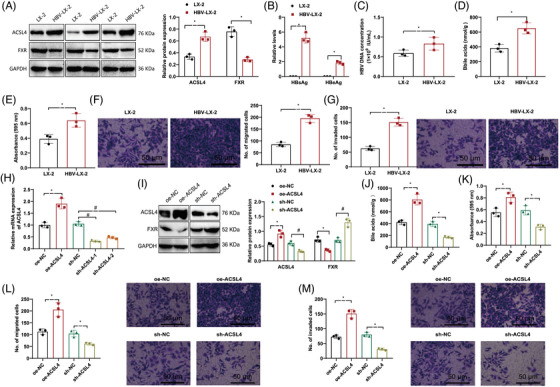
The regulation of ACSL4 in BAs and FXR in HCC cells. (A) Expression levels of ACSL4 and FXR in human hepatic stellate cells (LX‐2) and HBV‐infected LX‐2 cells detected by Western blot, with statistical significance between the two groups at *p* < 0.05. (B and C) Levels of HBsAg, HBeAg, and HBV DNA in LX‐2 cells and HBV‐infected LX‐2 cells detected by ELISA, with statistical significance between the two groups at *p* < 0.05. (D) BA levels in LX‐2 cells and HBV‐infected LX‐2 cells detected by mass spectrometry, with statistical significance between the two groups at *p* < 0.05. (E–G) Cell proliferation, migration, and invasive properties of HBV‐infected LX‐2 cells assessed by Transwell and MTT assays, with a scale of 50 µm, and statistical significance between the two groups at *p* < 0.05. (H) Overexpression and knockdown efficiency of ACSL4 in HBV‐LX‐2 cells detected by RT‐qPCR, compared with oe‐NC, # indicates comparison with sh‐NC, with statistical significance at *p* < 0.05. (I) Expression levels of ACSL4 and FXR in the supernatant of HBV‐LX‐2 cells detected by Western blot, compared with oe‐NC, # indicates comparison with sh‐NC, with statistical significance at *p* < 0.05. (J) BA levels in HBV‐LX‐2 cells detected by mass spectrometry, * indicates comparison with oe‐NC, # indicates comparison with sh‐NC, with statistical significance at *p* < 0.05. (K–M) Cell proliferation, migration, and invasive properties of HBV‐LX‐2 cells assessed by Transwell and MTT assays, with a scale of 50 µm. The cell experiments were independently repeated six times. FXR, farnesoid X receptor; HBV, hepatitis B virus; HCC, hepatocellular carcinoma.

The outcomes revealed an upsurge in ACSL4 protein expression in HBV‐LX‐2 cells as opposed to LX‐2 cells, while the expression of FXR was downregulated (Figure 4A). ELISA detected human hepatitis B virus surface antigen (HBsAg), hepatitis B e antigen (HBeAg), and HBV DNA levels in LX‐2 and HBV‐LX‐2 cells. It was observed that there was a rise in the presence of HBsAg, HBeAg, and HBV DNA in the HBV‐LX‐2 cells compared with LX‐2 cells (Figure 4B,C). The concentrations of BAs in HBV‐LX‐2 cells were detected using mass spectrometry analysis, and it was found that the BA levels were increased in HBV‐LX‐2 cells relative to LX‐2 cells (Figure 4D).

The results of Transwell and MTT assays demonstrate that in contrast with LX‐2 cells, HBV‐LX‐2 cells exhibit enhanced proliferation, migration, and invasion ability (Figure 4E–G). In addition, the impact of ACSL4 overexpression or downregulation in HBV‐infected LX‐2 cells was assessed through RT‐qPCR analysis. The results show that we successfully overexpressed or silenced ACSL4, with the most significant silencing efficiency observed in sh‐ACSL4‐1, paving the way for subsequent experiments (Figure 4H).

Through Western blot examination, it was observed that upregulation of ACSL4 led to a reduction in the levels of FXR, whereas downregulation of ACSL4 resulted in an elevation of FXR expression (Figure 4I). Mass spectrometry analysis showed that overexpression of ACSL4 increased the levels of BAs while silencing ACSL4 decreased the levels of BAs (Figure 4J). Transwell and MTT experiments showed that the upregulation of ACSL4 enhanced HCC cells’ proliferation, migration, and invasion abilities, while silencing ACSL4 diminished these abilities (Figure 4K–M).

The above results indicate that ACSL4's regulation of BAs and FXR pathways is instrumental in stimulating the proliferation, migration, and invasion behaviors of HCC cells.

### 2.5 FXR mediates M2 macrophage polarization to promote the emergence and progression of HCC

**FIGURE 5:**
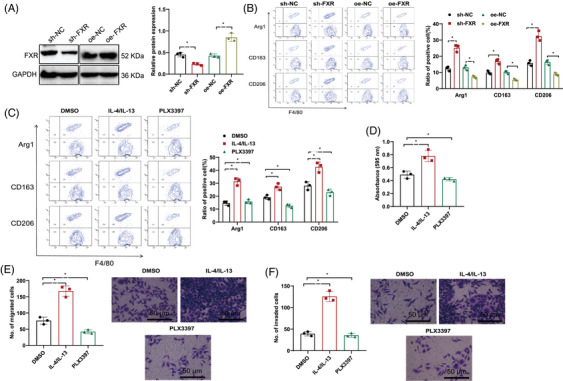
The FXR promotes the proliferation, migration, and invasion of HCC cells by influencing the polarization of M2 macrophages. (A) Western blot in the detection of FXR expression levels in the coculture system of HBV‐LX‐2 and THP‐1 macrophages. # represents comparison with oe‐NC, with significance level *p* < 0.05 compared with sh‐NC. (B) Flow cytometry in observing the expression of M2 macrophage polarization markers Arg1, CD163, and CD206 in the coculture of HBV‐LX‐2 and THP‐1 macrophages; (C) Flow cytometry for detecting the expression of M2 macrophage polarization markers Arg1, CD163, and CD206 in the coculture of HBV‐LX‐2 and THP‐1 macrophages. (D–F) Transwell and MTT assays for assessing the proliferation, migration, and invasion capacities of liver cancer cells in the coculture system of HBV‐LX‐2 and THP‐1 macrophages. The scale bar in the images indicates 50 µm, with *p* < 0.05 significance level. Each cell experiment was repeated six times. FXR, farnesoid X receptor; HBV, hepatitis B virus.

To examine the effects of FXR on the modulation of M2 macrophage polarization regarding the proliferative, migratory, and invasive potentials of hepatic cancer cells, we silenced or overexpressed FXR within the coculture setting with HBV‐LX‐2 and THP‐1 macrophages. Western blot analysis confirmed successful FXR silencing or overexpression (Figure 5A). Flow cytometry results demonstrated that FXR silencing led to an upregulation of indicators for M2 macrophage polarization, including Arg1, CD163, and CD206, whereas FXR overexpression led to their downregulation (Figure 5B).

Furthermore, to investigate the impact of M2 macrophage polarization on the cellular capacities for proliferation, migration, and invasion in liver cancer, we treated the HBV‐LX‐2 and THP‐1 macrophage coculture system with M2 polarization activators or inhibitors. The findings obtained through flow cytometry demonstrated that when contrasted with the DMSO control group, IL‐4/IL‐13, which are recognized as agents inducing M2 polarization, elevated the levels of markers associated with M2 macrophage polarization, including Arg1, CD163, and CD206, while PLX3397 (M2 polarization inhibitor) downregulated their expression (Figure 5C).

Transwell and MTT assays demonstrated that the proliferation, migration, and invasion capabilities of liver carcinoma cells were augmented by IL‐4 and IL‐13 contrasted with the DMSO control group, whereas PLX3397 weakened these abilities (Figure 5D–F).

These results suggest that silencing FXR can promote M2 macrophage polarization, which in turn enhances the proliferative, migratory, and invasive traits of liver malignancy cells.

### 2.6 ACSL4 regulates BA and FXR‐mediated M2 macrophage polarization, promoting the occurrence and development of HBV‐HCC

ACSL4 regulates BAs and FXR‐mediated M2 macrophage polarization to promote the progression of HBV‐HCC. Furthermore, following HBV infection, we utilized the OHO‐2 adhesive system to coculture LX‐2 cells and THP‐1 macrophages (HBV‐LX‐2 + THP‐1 macrophages).

**FIGURE 6:**
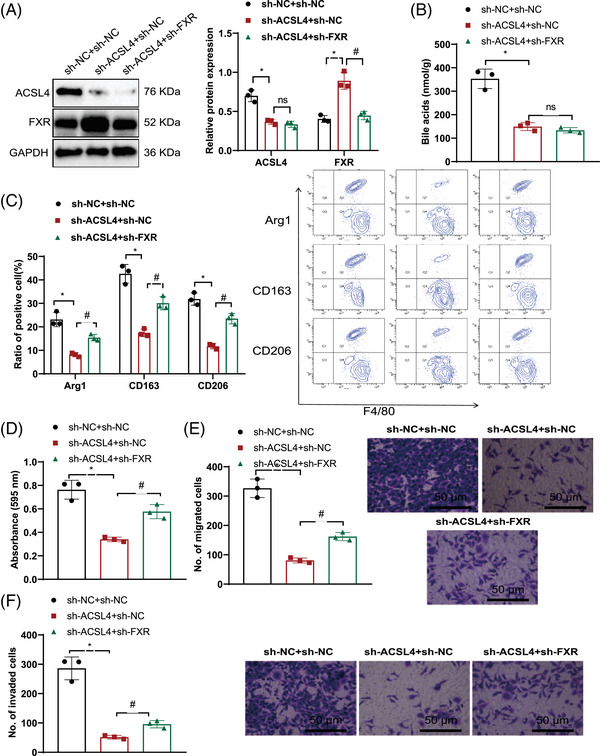
The effects of ACSL4 silence or FXR silence on the occurrence and development of HBV‐HCC. (A) In the coculture system of HBV‐LX‐2 cells and THP‐1 macrophages, silencing ACSL4 or silencing both ACSL4 and FXR simultaneously, Western blot was used to detect the expression levels of ACSL4 and FXR in liver cancer cells, compared with the sh‐NC + sh‐NC group, where # indicates comparison with the sh‐ACSL4 + sh‐NC group, with significance level *p* < 0.05. (B) In the coculture system of HBV‐LX‐2 cells and THP‐1 cells, silencing ACSL4 or silencing both ACSL4 and FXR simultaneously, mass spectrometry was used to measure the levels of BAs in liver cancer cells, compared with the sh‐NC + sh‐NC group, where ns indicates comparison with the sh‐ACSL4 + sh‐NC group, with significance level *p* < 0.05. (C) In the coculture system of HBV‐LX‐2 cells and THP‐1 cells, silencing ACSL4 or silencing both ACSL4 and FXR simultaneously, flow cytometry was used to detect the expression levels of M2 macrophage polarization markers Arg1, CD163, CD206, in comparison with the sh‐NC + sh‐NC group, where # indicates comparison with the sh‐ACSL4 + sh‐NC group, with *p* < 0.05 significance level. (D–F) In the coculture system of HBV‐LX‐2 + THP‐1 macrophages, silencing ACSL4 or silencing both ACSL4 and FXR simultaneously, Transwell assay was used to evaluate the proliferation, migration, and invasiveness of liver cancer cells. The scale bar in the images represents 50 µm, with comparison with the sh‐NC + sh‐NC group, where # represents comparison with the sh‐ACSL4 + sh‐NC group, at *p* < 0.05 significance level. The experiment was repeated six times. FXR, farnesoid X receptor; HBV, hepatitis B virus; sh‐NC, short hairpin negative control.

The analysis of Western blot results showed that in comparison with the sh‐NC + sh‐NC group, the sh‐ACSL4 + sh‐NC group exhibited downregulation of ACSL4 expression and upregulation of FXR expression. When juxtaposed with the sh‐ACSL4 + sh‐NC group, ACSL4 expression levels did not vary within the sh‐ACSL4 + sh‐FXR group, although a decrease in FXR expression was evident (Figure 6A). Mass spectrometry analysis of BA levels revealed decreased BA levels in the sh‐ACSL4 + sh‐NC group as opposed to the sh‐NC + sh‐NC group. The BA levels in the sh‐ACSL4 + sh‐FXR group did not change when compared with the sh‐ACSL4 + sh‐NC group (Figure 6B).

Assessment through flow cytometry indicated a decrease in the expression of M2 macrophage polarization markers Arg1, CD163, and CD206 in the sh‐ACSL4 + sh‐NC group when contrasted with the sh‐NC + sh‐NC group. Compared with the sh‐ACSL4 + sh‐NC group, the levels of Arg1, CD163, and CD206 expression was increased in the sh‐ACSL4 + sh‐FXR group (Figure 6C). MTT and Transwell experiments showed that in contrast with the sh‐NC + sh‐NC group, a reduction in cell proliferation, migration, and invasion was observed in the sh‐ACSL4 + sh‐NC experimental group. Amplified cell proliferation, migration, and invasiveness were observed in the sh‐ACSL4 + sh‐FXR group contrasted with the sh‐ACSL4 + sh‐NC group (Figure 6D–F).

The previously mentioned data points to the conclusion that ACSL4 regulates BAs and FXR‐mediated M2 macrophage polarization and promotes HCC cell proliferation, migration, and invasion.

### 2.7 Spontaneous mouse model of HBV‐HCC (HBs‐HepR mice)

The cell experiments of the spontaneous HBV‐HCC mouse model (HBs‐HepR mice) demonstrated the impact of overexpression or depletion of ACSL4 on the advancement of HBV‐HCC. Therefore, we constructed a spontaneous HBV‐HCC mouse model to verify further the effect of ACSL4 regulation on BA and FXR‐mediated macrophage polarization in the molecular mechanism of HBV‐HCC. HBs‐Tg mice are transgenic mice that contain the human HBsAg gene. HBsAg is one of the leading indicators of HBV infection. We isolated hepatocytes (HBsAg+ liver cells) from HBs‐Tg mice and transferred these cells to immune‐competent Fah‐deficient mice via splenic injection for liver cell reconstitution. Ultimately, we successfully generated HBs‐HepR mice.^49^, ^50^

After constructing the HBs‐HepR mouse model, we found that relative to the control group, the ALT and AST serum levels in the model group mice were elevated throughout the injection process (Figure S2A,B). Furthermore, the levels of HBsAg, HBeAg, and HBV‐DNA were upregulated (Figure S2C,D). H&E staining showed diffuse necrotizing inflammation in liver tissue sections of the model group mice, with visible infiltrates of mononuclear cells and deep infiltration of liver cells (Figure S2E). Evaluation of liver fibrosis involved the application of Sirius Red staining to examine collagen deposition/accumulation. The model group of mice exhibited severe fibrosis (Figure S2F). Therefore, we assessed the quantities of CD8+ T cells and the expression levels of IL‐2, TNF‐α, and IFN‐γ in HBs‐HepR mice.

Immunohistochemistry and ELISA outcomes indicated that, when contrasted with the control group, the quantity of CD8+ T cells in the model group of mice increased, while the levels of IL‐2, TNF‐α, and IFN‐γ expression showed a reduction (Figure S2G‐H). At 40 weeks of injection, we observed large liver tumor nodules (≥3 mm^2^) on the surface of the liver in the model group of mice, while only four mice in the control group showed 1 or 2 smaller tumor nodules (≤2 mm^2^). The cumulative quantity of tumor nodules in the liver increased after H&E staining of liver sections (Figure S2I).

The above results indicate that by transferring hepatocytes (HBsAg+ liver cells) to immunocompetent Fah‐deficient mice via splenic injection, liver cell reconstruction could be induced, resulting in pathological liver damage, fibrosis and HCC, suggesting that we successfully constructed a spontaneous HBs‐HepR mouse model.

### 2.8 ACSL4 regulates BA and FXR‐mediated M2 macrophage polarization in the context of HBV‐HCC progression, as confirmed by in vivo animal experiments

Experimental studies conducted on animals have validated that ACSL4 regulates BA and FXR‐mediated M2 macrophage polarization, which contributes to the progression of HBV‐HCC. To further investigate the effects of ACSL4 regulation on BA and FXR‐mediated macrophage polarization in the molecular mechanisms of HBV‐HCC, we treated HBs‐HepR mice by silencing ACSL4 alone or silencing both ACSL4 and FXR simultaneously.

**FIGURE 7:**
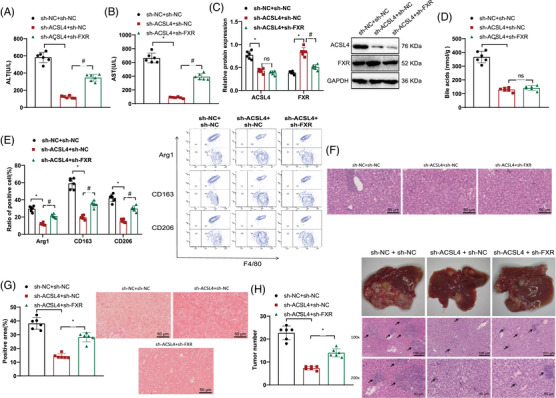
Regulation of BAs and FXR‐mediated M2 macrophage polarization by ACSL4 in HBV‐HCC development. (A and B) Levels of alanine transaminase (ALT) and aspartate transaminase (AST) in mouse serum measured by ELISA, *n* = 6. Comparative analysis: sh‐NC + sh‐NC versus sh‐ACSL4 + sh‐NC, *p* < 0.05. (C) Protein expression levels of ACSL4 and FXR in mouse liver tissue detected by Western blot, *n* = 6. Comparative analysis: sh‐NC + sh‐NC versus sh‐ACSL4 + sh‐NC, *p* < 0.05. (D) BA levels in mouse liver tissue measured by mass spectrometry analysis, n = 6. Comparative analysis: sh‐NC + sh‐NC versus sh‐ACSL4 + sh‐NC, ns, *p *> 0.05. (E) Expression of M2 macrophage polarization markers Arg1, CD163, and CD206 detected by flow cytometry assay, *n* = 6. Comparative analysis: sh‐NC + sh‐NC versus sh‐ACSL4 + sh‐NC, *p* < 0.05. (F) Histopathological changes in mouse liver observed by H&E staining, scale bar: 50 µm. (G) Fibrosis analysis of collagen deposition in mouse liver using Sirius Red staining, *n* = 6. Comparative analysis: sh‐NC + sh‐NC versus sh‐ACSL4 + sh‐NC, scale bar: 50 µm, *p* < 0.05. (H) Nodule formation in mouse liver observed by H&E staining, black arrows indicate nodules, scale bar: 100 and 50 µm, *n* = 6. Comparative analysis: sh‐NC + sh‐NC versus sh‐ACSL4 + sh‐NC, *p* < 0.05. ALT, alanine transaminase; AST, aspartate transaminase; FXR, farnesoid X receptor; H&E, hematoxylin and eosin.

ELISA results showed that in contrast with the sh‐NC + sh‐NC group, the serum ALT and AST concentrations experienced a decline in the sh‐ACSL4 + sh‐NC group of mice. However, as opposed to the sh‐ACSL4 + sh‐NC group, the serum showed heightened levels of ALT and AST in the sh‐ACSL4 + sh‐FXR group of mice (Figure 7A,B).

Protein levels of ACSL4 in the liver tissue of mice were found to be lower in the sh‐ACSL4 + sh‐NC group when contrasted with the sh‐NC + sh‐NC group according to Western blot analysis, while FXR protein levels were increased. In comparison with the sh‐ACSL4 + sh‐NC group, no disparity was observed in the ACSL4 protein levels of mouse liver tissue in the sh‐ACSL4 + sh‐FXR group, but FXR protein levels were decreased (Figure 7C).

Analysis via mass spectrometry revealed a decrease in the quantity of BAs present in the liver tissue of mice within the sh‐ACSL4 + sh‐NC experimental group in contrast to the sh‐NC + sh‐NC control group. There was no change in BA levels in the liver tissue of mice in the sh‐ACSL4 + sh‐FXR group as opposed to the sh‐ACSL4 + sh‐NC group (Figure 7D).

Flow cytometry results showed that the sh‐ACSL4 + sh‐NC group showed decreased expression of M2 macrophage polarization markers Arg1, CD163, and CD206 in liver samples, whereas the sh‐NC + sh‐NC group did not exhibit such reductions; in contrast with the sh‐ACSL4 + sh‐NC group, the expression levels of M2 macrophage polarization markers Arg1, CD163, and CD206 in the liver tissues of mice in the sh‐ACSL4 + sh‐FXR group were upregulated (Figure 7E).

H&E staining results showed that when juxtaposed with the sh‐NC + sh‐NC group, the cellular inflammation and infiltration in the liver tissue of mice in the sh‐ACSL4 + sh‐NC group were alleviated. The sh‐ACSL4 + sh‐FXR group exhibited elevated levels of cellular inflammation and infiltration in the liver tissue, in contrast to the sh‐ACSL4 + sh‐NC group's presentation (Figure 7F).

Sirius Red staining analysis revealed a decrease in collagen deposition/fibrosis in the liver tissue of mice in the sh‐ACSL4 + sh‐NC group contrasted with the sh‐NC + sh‐NC group and an increase in collagen deposition/fibrosis in the liver tissue of mice in the sh‐ACSL4 + sh‐FXR group as opposed to the sh‐ACSL4 + sh‐NC group (Figure 7G).

The tumor volume and total number of tumor nodules in the liver tissue of mice in the sh‐ACSL4 + sh‐NC group were reduced when juxtaposed with the sh‐NC + sh‐NC group, while the tumor volume and total number of tumor nodules in the liver tissue of mice in the sh‐ACSL4 + sh‐FXR group were increased in comparison with the sh‐ACSL4 + sh‐NC group (Figure 7H).

In summary, the results demonstrate that ACSL4 regulates BAs and FXR‐mediated M2 macrophage polarization, which is implicated in the onset and progression of HBV‐HCC.

## 3 DISCUSSION

This study highlighted the vital function of ACSL4 in the occurrence and progression of hepatitis HBV‐HCC. Through bioinformatics analysis and clinical sample testing, ACSL4 was determined to be a key gene affecting HBV‐HCC. This discovery aligns with previous research on the regulatory role of ACSL4 in other cancer types but, for the first time, highlights the central role of ACSL4 in HBV‐HCC.^51^ This finding provides a new direction for understanding the molecular mechanisms of liver cancer and potentially identifies a target for future therapies.

Our research revealed elevated levels of BAs in liver tissues of HBV‐HCC patients. Evidence from in vitro and in vivo studies substantiated that silencing ACSL4 could reduce BA levels, thus improving the progression of HBV‐HCC. While previous studies have focused on the link between BA metabolism and liver diseases, the specific interaction and regulatory mechanisms between ACSL4 and BAs remain largely unknown.^52^ Our findings fill this knowledge gap, revealing a novel regulatory pathway.

Another significant discovery of our study is that ACSL4 influences the occurrence and progression of HBV‐HCC through FXR‐mediated polarization of M2 macrophages. M2 macrophages play a crucial role in promoting angiogenesis, tumor growth, immune evasion, and therapeutic resistance in HCC.^53^ This mechanism has not been extensively explored in previous studies. Our experimental data indicate that the upregulation of ACSL4 and M2 macrophage polarization markers (Arg1, CD163, CD206) have a significant correlation with the development of HBV‐HCC, while the expression of M1 markers (CD86, CD80, CD11c) is significantly downregulated (Figure S3). This new finding presents a unique viewpoint on interpreting the tumor microenvironment and immune response in liver cancer.

Historically, research on BA metabolism and HBV‐HCC has primarily focused on in vitro observations, lacking a comprehensive understanding of these molecular mechanisms in living systems.^54^ By utilizing the HBs‐HepR mouse model, our in vivo experimental evidence further strengthens the reliability and applicability of our findings.

In conclusion, we propose that ACSL4 regulates BAs and FXR‐mediated polarization of M2 macrophages, thereby influencing the occurrence and progression of HBV‐HCC. This study reveals the critical role of ACSL4 in governing BAs and promoting FXR‐mediated M2 macrophage polarization in HBV‐HCC, unveiling a novel molecular regulatory network.^2^ Through bioinformatics analysis and in vitro and in vivo experiments, our study establishes a comprehensive molecular mechanism model that may propose an innovative theoretical groundwork for tackling liver cancer prevention and therapy.

It is worth noting the association between ACSL4 and ferroptosis. As early as 1997, Kang et al.^55^ found that AA and Eicosapentaenoic acid (20:5) are major substrates of ACSL4. Knockout of the Acsl4 gene specifically in adipocytes in mice contributed to a marked decline in the amount of arachidonic acid or docosapentaenoic acid (22:5) in PL and an increase in LA,^56^ highlighting the crucial role of ACSL4 in ferroptosis regulation.^57^ We conducted a simple test by staining HBV‐LX‐2 cells with FerroOrange and found that ferrous ion accumulation showed a notable decrease in the ACSL4 silenced group when juxtaposed with the control group (Figure S4). While the relationship between iron death and the occurrence and development of HBV‐HCC warrants further exploration due to experimental limitations, we plan to delve deeper into this in future studies.

Clinically, the treatment of HBV‐HCC remains a challenge.^3^, ^9^, ^58^ Our study suggests that silencing ACSL4 can enhance the onset and evolution of HBV‐HCC, potentially guiding the development of new drug targets. Furthermore, analyzing BA levels, M2 macrophage polarization markers, and FXR expression could serve as diagnostic and prognostic indicators for HBV‐HCC. A point to be emphasized is that the samples used in this study might differ from the general population and may not fully reflect the situation of all HBV‐HCC patients. While validations were performed using in vitro cell lines and mouse models, differences may exist in human contexts, necessitating further clinical trials to confirm these findings. Although our study primarily focuses on the regulatory mechanisms of ACSL4 and BAs, there may be other unknown regulatory networks that warrant further investigation.

Despite achieving significant breakthroughs in many aspects, there are still several questions worth exploring further. These include the specific interaction mechanisms between ACSL4 and BA metabolism, detailed mechanisms of FXR action, and the deep‐seated mechanisms of ACSL4 regulating M2 macrophage polarization. Addressing these questions will deepen our understanding of the complex molecular mechanisms of HBV‐HCC and aid in the development of more effective treatment methodologies.

## 4 MATERIALS AND METHODS

### 4.1 Public database chip data acquisition

Data on HBV infection leading to LIHC was retrieved from the GEO database, specifically from chip datasets GSE121248 and GSE55092, with sample sizes of 37 normal and 70 HBV‐HCC, and 26 normal and 37 HBV‐HCC, respectively.

Analysis of differential gene expression in these datasets was executed through the application of the “limma” package in the R software using standard control samples as references. Identification of DEGs was achieved with an adjusted *p* value filter threshold of <0.05. For visual representation, creation of expression heatmaps and volcano plots representing the DEGs was accomplished through the utilization of the “pheatmap” package in the R programming language. To identify common DEGs between the GSE121248 and GSE55092 datasets, the “VennDiagram” package in R software was employed, resulting in a comprehensive intersection of differentially expressed genes from both chip datasets.

The chip datasets GSE121248 and GSE55092 were subjected to data correction, and data merging was performed using a Perl script. Perform differential analysis on the merged dataset and conduct PPI analysis for the differentially expressed genes using the STRING website for further research.^59^ Subsequently, the merged genes were subjected to GSEA enrichment analysis using GSEA_4.3.2 software to identify metabolic‐related pathways.

### 4.2 Examination of infiltration by immune cells

Utilization of the CIBERSORT algorithm was implemented on datasets retrieved from the GEO database, using R software and known expression profiles of 22 immune cell‐specific genes. The result was the relative abundance of varied immune cell types in the tissue samples. Filter the matrix data of diverse immune cell types in the organizational samples using Perl scripts, with the filtering condition as *p*valueOpt < 0.05, followed by visualization analysis.^60^

### 4.3 Collection of samples from HBV‐HCC patients

Cancer and adjacent standard tissues were collected from 20 HBV‐HCC patients, aged 40−65 years, averaging 55 years, undergoing surgery at our hospital. None received presurgery chemotherapy or radiotherapy. The adjacent normal tissues, obtained from the same patients, were situated at a distance exceeding 5 cm from the tumor margin. The gathered tissues were divided in two: one part was promptly preserved in liquid nitrogen, and the other was preserved in a 10% formaldehyde solution and subsequently encased in paraffin for sectioning. This study received approval from Shenzhen People's Hospital (NO. XYZ‐2023‐04‐21), the Second Clinical Medical College, Jinan University's Clinical Ethics Committee and informed consent was obtained from the patients, aligning with the Helsinki Declaration's provisions.^61^

### 4.4 Cell culture and transfection

Cultivation of the human normal hepatic stellate cell line LX‐2 (SNL‐206) was carried out in DMEM (11965092, USA) containing 10% fetal bovine serum (FBS500‐S; AusgeneX) under standard conditions of 37°C and 5% CO_2_. The complete genome of the HBV C2 subtype and the 1.2‐fold gene sequence of the pAAV/HBV1.2 plasmid was constructed.

pAAV‐MCS (kl‐zl‐0986‐01) was used as the vector to construct the pAAV/HBV1.2C2 recombinant using gene synthesis and homologous recombination (completed by BGI Genomics and Huada Gene Technology Co., Ltd.). Extract plasmids using the high‐purity Midiprep Kit (ZP104‐1; Zoman). To transfer pAAV/HBV1.2C2 into LX‐2 cells, lipofectamine transfection reagent (L3000015; Invitrogen™) was utilized in the experiment. The supernatant was collected after 36 h. HBsAg expression in the cell culture supernatant of pAAV/HBV1.2C2‐transfected cells using HBsAg diagnostic kit (TL16962; China) was detetcted.^62^, ^63^

LX‐2 cells infected with HBV were divided into several groups according to different treatment methods: oe‐NC group (overexpression lentiviral negative control group), oe‐ACSL4 group (overexpression lentiviral ACSL4 group), sh‐NC group (shRNA lentiviral negative control group), and sh‐ACSL4 group (shRNA ACSL4 lentiviral group). See Table S1 for knockdown sequences. The lentiviruses expressing overexpression and repression were constructed and provided by Shanghai Genomics, a Genemill Biological Engineering Co. Ltd. subsidiary in Shanghai, China.

LX‐2 cells in the state of logarithmic growth were accumulated and then resuspended with a density of 5 × 10^4^ cells/mL. Placed in a six‐well tray (2 mL per well), the suspension underwent overnight incubation at a temperature of 37°C. The addition of recombinant lentivirus (at a final concentration of 1 × 10^8^ TU/mL), with either silenced or overexpressed genes, took place in each well followed by a 24‐h postinfection incubation period. The infection efficiency of GFP was observed using a fluorescence microscope, and cells exhibiting high infection efficiency were singled out for additional experimentation. This process was reiterated thrice.^62^

### 4.5 Cooperative education system

The HBV‐LX‐2 and THP‐1 macrophage (FSX6345) cell lines were cultivated as follows: THP‐1 macrophage cells were cultivated in RPMI‐1640 medium containing 10% FBS, 0.05 mM β‐mercaptoethanol, and 1% P/S (CM‐0233). These cells were kept at 37°C in a 5% CO_2_ setting. To induce differentiation into macrophages, THP‐1 macrophage cells underwent treatment with 100 ng/ml PMA (Sigma–Aldrich, USA) for a 24‐h period.

Silence or overexpression of FXR was performed in HBV‐LX‐2 cells, followed by coculturing with THP‐1 macrophage cells in a six‐well Transwell coculture system (FCP060; BeyoGold). The groups were divided as follows: sh‐NC group (silenced lentivirus control group), sh‐FXR group (silenced FXR lentivirus group), oe‐NC group (overexpression lentivirus control group), and or‐FXR group (overexpression FXR lentivirus group).

After adding M2 polarization activators or inhibitors to THP‐1 macrophage cells, coculture with HBV‐LX‐2 was performed with the following groups: DMSO group (control group, added with DMSO solution) (MFCD00002089), IL‐4 (20 ng/mL, dissolved in DMSO solution)/IL‐13 group (20 ng/mL, solution prepared in DMSO) (M2 polarization activators) (two activator products numbers SRP4137 and SRP4166), and PLX3397 (10 mmol/L, dissolved in DMSO solution) (M2 polarization inhibitor) (A15520).^64^

After silencing ACSL4 alone and simultaneously silencing ACSL4 and FXR in HBV‐LX‐2 cells, coculturing was performed with THP‐1 macrophage macrophage cells, and groups were as follows: sh‐NC + sh‐NC group (silencing lentiviral control group + silencing lentiviral control group), sh‐ACSL4 + sh‐NC group (silencing lentiviral ACSL4 + silencing lentiviral control group), and sh‐ACSL4 + sh‐FXR group (silencing lentiviral ACSL4 + silencing lentiviral FXR group). The FXR silencing sequence is shown in (Table S1).^65^, ^66^ The transfection method is the same as mentioned above.

### 4.6 Transwell experiments

The upper chamber of Transwell was filled with THP‐1 macrophages (2 × 10^4^ cells per well) and RPMI1640 (R8758) medium. The lower well of the Transwell contained HBV‐LX‐2 cells cultured in DMEM medium supplemented with 10% FBS (FBS500‐S; AusgeneX). After culturing for 24 h at 37°C, the cells in the upper chamber were gently swiped using a cotton swab. Then, 4% glutaraldehyde was applied to fix the cells, use 0.1% crystal violet for staining, then wash with PBS three times. Finally, the migration and invasion of HCC cells using an optical microscope were observed.^62^, ^67^

### 4.7 MTT proliferation experiment

The experiment involved introducing 5 × 10^3^ HCC cells into a 24‐well plate. Initially, each well was supplemented with 500 µL of 0.5 mg/mL MTT solution (T10182; Shangbao) and cultured at 37°C, 5% CO_2_, for a duration of 2−4 h. Following this, the solution was discarded and 200−500 µL of DMSO were included in every well. After agitating on the oscillator for 20 min, spectrophotometric analysis was employed to assess cell absorbance at 570 nm. Every sample was replicated in a set of three wells.^68^

### 4.8 Spontaneous HBV‐HCC mouse model acquisition and other treatments

HBV transgenic mice C57BL/6J‐TgN (AlblHBV) 44Bri (HBs‐Tg) and Fah^−/−^ mice on a C57BL/6J background, as well as normal C57BL/6J mice, were sourced from Beijing Vecto Biotechnology Co., Ltd. SPF‐grade animal facilities are utilized for housing all mice, ensuring they are situated in an environment with humidity levels of 60–65% and temperatures ranging between 22 and 25°C. The trial commenced subsequent to a week of acclimatization to the feeding regimen, during which the mice's health status was assessed ahead of the commencement of the experiment. The experimental procedures on animals adhere to the protocols set forth in the “Guide for the Care and Use of Laboratory Animals” and have been authorized by the Institutional Animal Ethics Committee.

The Fah^−/−^ mice were treated and divided into two groups, each containing six mice. The groups were as follows: control group (standard group) and model group (treated with HBsAg+ liver cells). HBsAg+ hepatocytes, extracted from HBs‐Tg mice, were transplanted into immunocompetent receptor Fah^−/−^ mice in the model group. This transfer successfully generated HBs‐HepR mice.^49^

The isolation and culture of HBsAg+ liver cells involved several detailed steps. Initially, HBs‐Tg mice were anesthetized by injecting 5% pentobarbital into their peritoneal cavity. Once anesthetized, the mice were positioned prone on the operating table, and their livers were surgically removed under sterile conditions. The excised livers were then thoroughly rinsed with cold PBS to eliminate any remaining blood. Following this, the livers were minced into smaller sections and subjected to digestion with a buffer solution infused with digestive enzymes (3‐0002, OptiTDS™; Qiagen) for cell segregation. The separated cells underwent multiple cold PBS washes to remove any leftover enzymes. After discarding dead and fragmented cells, cultivation of primary cells was conducted in DMEM medium (11965092; USA) enriched with 10% FBS (FBS500‐S; AusgeneX) at a consistent 37°C temperature under 5% CO_2_ conditions.

Subsequently, 1 × 10^6^ hepatocyte suspension was injected into the spleen of Fah^−/−^ mice using a syringe to ensure the cells were accurately injected into the spleen rather than into the splenic vessels. Inject liver cells isolated from normal C57BL/6J mice into the control group. At 14 weeks, evaluation was conducted using methods such as testing the concentrations of ALT, AST, and HBV‐DNA in blood samples, as well as assessing the count of CD8+ T cells and the expression levels of IL‐2, TNF‐α, and IFN‐γ in tissue samples, the pathological changes in mouse liver, the fibrosis situation of collagen deposition/accumulation, and the condition of tumor nodules.^10^, ^49^

The HBs‐HepR mice were managed and classified into three groups, with six mice allocated to each group. The animal grouping is as follows: sh‐NC + sh‐NC group (silencing lentivirus control + silencing lentivirus control), sh‐ACSL4 + sh‐NC group (silencing lentivirus ACSL4 + silencing lentivirus control), sh‐ACSL4 + sh‐FXR group (silencing lentivirus ACSL4 + silencing lentivirus FXR). The titer of the slow virus is 1 × 1011 PFU. It was purchased from Jima Biology. The injection volume per mouse via the tail vein is approximately 50 µL. The therapy is given biweekly for a period of 7 weeks continuously.

Blood samples were gathered from groups of six mice at the 40‐week mark for the assessment of ALT and AST enzyme concentrations. Liver tissue was also collected to assess BA levels, pathological changes, fibrosis, and nodules.^10^, ^69^

### 4.9 RT‐qPCR

After isolation using TRIzol (15596026; ThermoFisher, USA), the total RNA was converted into cDNA by employing the High‐Capacity cDNA Reverse Transcription Kit (4368813; Invitrogen, USA). Using GAPDH as an internal reference, we conducted RT‐PCR experiments on the ABI7500 quantitative PCR instrument (ABI, USA) using the SYBR Green reagent kit (K0222; Thermo Scientific). The PCR reaction mixture undergoes PCR amplification on a real‐time fluorescence quantitative PCR device. Utilizing the 2^−ΔΔCt^ technique, the ultimate data underwent comprehensive analysis. The primer sequences are available in Table S2, with the primers being manufactured by Shanghai Biotech Co., Ltd.^70^

### 4.10 Western blot

The extraction of total protein from cell groups was carried out with the protein extraction kit (ab113476; Abcam) and the protein concentration in the samples was assessed using the BCA assay kit (BCA1; Sigma–Aldrich). We prepared a 10% SDS‐PAGE gel (P0012A; Biyun Biosciences Institute, Shanghai, China). The protein samples, 50 µg in each well, were loaded and electrophoresed under constant voltages of 80 and 120 V for 2 h. Then, we transferred the protein onto a PVDF membrane (ISEQ00010; Millipore, Billerica, MA, USA) employing a wet transfer method and subjected it to a constant current of 250 mA for 90 min. The PVDF membrane was treated by immersing it in TBST buffer with an addition of 5% skim milk powder for a time period of 2 h. The blocking solution was drained, washed once with TBST, and the primary antibody was added and incubated overnight at 4°C (refer to antibody details listed in Table S3). The membrane was then washed three times with TBST, each time for 10 min. Next, the goat anti‐rabbit IgG was added to secondary antibody conjugated with horseradish peroxidase (HRP), ab6721, and incubated at ambient temperature for 60 min, and was washed three times with PBST, each time for 10 min. Finally, the membrane was immersed into the ECL reaction solution (MA0186‐3; Meilunbio) for color development and was exposed in a dark box for autoradiography. Using GAPDH as a reference, the calculation of the protein's relative expression level involves assessing the grayscale value of the target band relative to the reference band.^71^

### 4.11 MS testing total BA levels

#### 4.11.1 Cell preparation

Preparation of cell samples started with the removal of the culture medium from the dish. The next step involved two washes of the cells with 1 mL of cold PBS. After washing, 750 µL of cold methanol and 25 µL of IS solution (140‐111‐081; USA) were added to each dish, allowing it to stand for 5 min. Cells were dislodged from the surface using a scraper. The resulting extract was collected in a clean tube. An additional 250 µL of chilled methanol was added to the dish, mixed with the previous liquid to optimize recovery. The transfer of the supernatant to a fresh tube occurred postcentrifugation at 10,000×*g* for 10 min at 4°C. The samples were then evaporated to dryness and stored at −20°C until further analysis.

#### 4.11.2 Liver tissue preparation

For liver tissue preparation, a frozen tissue sample weighing between 30 and 100 mg was placed in a 2‐mL tube containing CK14 ceramic beads. The precise weight of the tissue was recorded. Corresponding to every 100 mg of tissue, 600 µL of cold methanol and 200 µL of IS solution were added. The tissue was then homogenized twice using a Precellys 24 dual system with a Criolys cooler set to 6000 rpm at 4°C for 25 s each cycle. Post homogenization, centrifugation of the sample was conducted at a force of 3000×*g* for a period of 5 min at a temperature of 4°C. Subsequently, the supernatant was carefully transferred to a fresh tube. An extra 400 µL of cold methanol was added for every 100 mg of tissue, and the extraction process was repeated. The supernatants from the two extraction cycles were combined. A total of 150 µL of this combined sample was placed in a new tube (remaining samples could be stored at −80°C), evaporated to dryness, and stored at −20°C until analysis, as per the methods described in PMID: 25270933.

Quantitative analysis by MS was conducted using the Shimadzu LC‐30A CL UPLC (LC‐30A). The MS analysis is carried out with the LCMS‐8050 CL triple quadrupole instrument incorporating an electrospray ionization source. The chromatographic separation procedure involved the use of a Phenomenex Kinetex C18 column measuring 3.0 × 100 mm and having a particle size of 2.6 µm. After separation, the samples are ionized using electrostatic spray ionization in negative ion mode and are subsequently recognized utilizing multiple reaction monitoring techniques. The instrumental settings for the MS analysis were as described below: the nebulizer flow rate is 3.0 L/min, the heating flow rate is 10.0 L/min, the interface temperature is 400°C, the desolvation line (DL) temperature is 300°C, the heating block temperature is 500°C, and the drying flow rate is 10.0 L/min. The overall quantity of BA present in the sample was calculated by summing the concentrations of all identified and quantified BAs. The selection of 42 BA standards in this research encompasses cholic acid, lithocholic acid, taurocholic acid, chenodeoxycholic acid, deoxycholic acid, taurolithocholic acid, hyodeoxycholic acid, hyolithocholic acid, and other BAs, purchased from Sigma–Aldrich (330707; Avanti). A sample injection volume of 5 µL is administered, maintaining a flow rate of 0.3 mL/min for optimal performance.^72^

### 4.12 Intracellular ferrous ion detection

Intracellular total ferrous ions in living cells were detected using FerroOrange (10 µM). Following treatment with experimental compounds for a specified period, cells in 24‐well culture plates were stained with FerroOrange in HBSS at 37°C, for 30 min. After three washes, intracellular FerroOrange fluorescence was captured using fluorescent microscopy.

### 4.13 ELISA detection

The expression levels of HBsAg (AD20522; ADANTI, China), HBeAg (AD21696; ADANTI), HBV‐DNA (AD21848; ADANTI), IL‐2 (AD40139; ADANTI), TNF‐α (AD40104; ADANTI), and IFN‐γ (AD40155; ADANTI) were detected in the supernatant of the culture medium and mouse liver tissues using ELISA kits. The samples and control samples were added separately to different wells, incubated with the enzyme‐linked antibody (Ab‐HRP) (NRE25‐0.5 mL) at 37°C for 1 h, and washed with PBST five times. The reaction was halted after the incubation of 100 mL substrate solution in each well for 15 min. Analyzing the absorbance at 450 nm was carried out with a microplate reader (BIO‐RAD, USA).^63^

### 4.14 Flow cytometry

The detection of macrophage polarization markers Arg1 (17‐3697‐82, 1.0 µg/test), CD163 (12‐1631‐82, 0.25 µg/test), and CD206 (17‐2061‐82, 0.25 µg/test) was performed utilizing flow cytometry. The macrophages (1 × 10^6^) were stained following the manufacturer's guidelines, using the F4/80 monoclonal antibody (MA1‐91124, 1/100; Thermo Fisher). This staining procedure assisted in the effective marking of the macrophages for the intended analysis. The collected data were subsequently analyzed, deploying the BD FACSMelody™ 4‐Way Cell Sorter from BD.^70^

### 4.15 Biochemical testing

As per the instructions outlined by the manufacturer, commercially available reagent kits were utilized for assessing the concentrations of ALT (BA1561; Shangbao) and AST (LZ‐S01109; Lianzhu).^73^

### 4.16 Histopathological analysis

Liver tissues were meticulously harvested from each group of mice and processed into 5 µm sections for subsequent staining. The tissue sections were initially subjected to hematoxylin (517‐28‐2; JiSheng Biotech) staining at room temperature for 10 min for the H&E staining process, followed by a rinse with running water lasting 30−60 s. Differentiation was conducted by immersing the sample in 1% hydrochloric acid solution for 30 s, followed by a subsequent rinse under tap water for 5 min. Eosin staining was applied at ambient temperature for 1 min. The sections were then sequentially dehydrated with graded alcohol (70, 80, 90, 95, and 100%) for 1 min each. After immersion in xylene with stone coal for 1 min and two rounds of 1‐min clarification, the sections were sealed with neutral resin. Poststaining, examination of liver tissue sections was conducted with a light microscope (BX50; Olympus) for analyzing morphological changes or counts across different groups, aiming to discern specific pathological changes in the liver.^74^

*Sirius Red staining protocol*: Liver tissue samples were exposed to a saturated solution comprising 0.1% Sirius Red and 0.1% picric acid for a duration of 1 h to facilitate the staining process. Collagen protein's distribution area was measured through the application of Image Pro Plus software (Media Cybernetics, Bethesda, MD), where four continuous images (magnification ×40) were captured for each slide.^69^

Immunohistochemical analysis of mouse liver tissue embedded in paraffin was performed by using CD8 antibody (ab217344) at a dilution of 1/2000, followed by labeling with goat anti‐rabbit IgG H‐1L (HRP) (ab127896) at a dilution of 1/1000.^75^, ^76^

### 4.17 Statistical analysis

The analysis of all data was conducted utilizing Statistical Package for the Social Sciences 26.0 software (IBM, USA). Continuous variables were expressed as mean ± standard deviation. The comparison of differences between two groups was carried out with a *t*‐test, whereas differences among multiple groups were analyzed through one‐way ANOVA. When *p* < 0.05, it indicates that the difference is statistically significant.

## AUTHOR CONTRIBUTIONS

Wenbiao Chen, Huixuan Xu, and Liliangzi Guo designed the study. Fengping Zheng, Jun Yao, Lisheng Wang, and Liliangzi Guo collated the data, designed and developed the database, carried out data analyses, and produced the initial draft of the manuscript. Wenbiao Chen and Liliangzi Guo contributed to drafting the manuscript. All authors have read and approved the final submitted manuscript.

## CONFLICT OF INTEREST STATEMENT

The authors declare no conflict of interest.

## ETHICS STATEMENT

This study received approval from Shenzhen People's Hospital, The Second Clinical Medical College, Jinan University's Clinical Ethics Committee (AUP‐240423‐CWB‐309‐01) and informed consent from the patients, aligning with the Helsinki Declaration's provisions. All animal experiments are conducted according to the “Guide for the Care and Use of Laboratory Animals” and have obtained approval from the Institutional Animal Ethics Committee of Shenzhen People's Hospital, The Second Clinical Medical College, Jinan University (2024‐249‐01).

## Supporting information

Supporting Information

## Data Availability

The original contributions presented in the study are included in the article/supplementary materials. Any further inquiries can be directed to the corresponding author.
