# Supplementary material for: Role of ACSL4 in modulating farnesoid X receptor expression and M2 macrophage polarization in HBV‐induced hepatocellular carcinoma
Source: MedComm (2020). 2024 Sep 12;5(9):e706. doi: 10.1002/mco2.706 (PMC11391271; doi:10.1002/mco2.706)
Supplement: Supplementary file 1 — Supporting Information [file MCO2-5-e706-s001.docx]

**Role of ACSL4 in Modulating Farnesoid X Receptor Expression and M2 Macrophage Polarization in HBV-Induced Hepatocellular Carcinoma**

**Running Title: ACSL4 and HBV-HCC Mechanisms**

**Wenbiao Chen^1*^, Huixuan Xu^2^, Liliangzi Guo^1^, Fengping Zheng^3^, Jun Yao^1^, Lisheng Wang^1*^**

^1^Department of Gastroenterology, Shenzhen People's Hospital, The Second Clinical Medical College, Jinan University; The First Affiliated Hospital, Southern University of Science and Technology, Shenzhen, 518020, China.

^2^Department of Rheumatology and Immunology, The Second Clinical Medical College, Jinan University (Shenzhen People's Hospital), Shenzhen, 518020, China.

3 Shenzhen Peking University-The Hong Kong University of Science and Technology Medical Center, Peking University Shenzhen Hospital, Shenzhen, Guangdong, 518036, China.

**^*^Corresponding author:** Wenbiao Chen and Lisheng Wang，

Department of Gastroenterology, Shenzhen People's Hospital, The Second Clinical Medical College, Jinan University; The First Affiliated Hospital, Southern University of Science and Technology, No. 1017, Dongmen North Road, Luohu District, Shenzhen, Guangdong Province, 518020, China.

1. mail: chanwenbiao@sina.com (Wenbiao Chen); wangls168@163.com (Lisheng Wang).


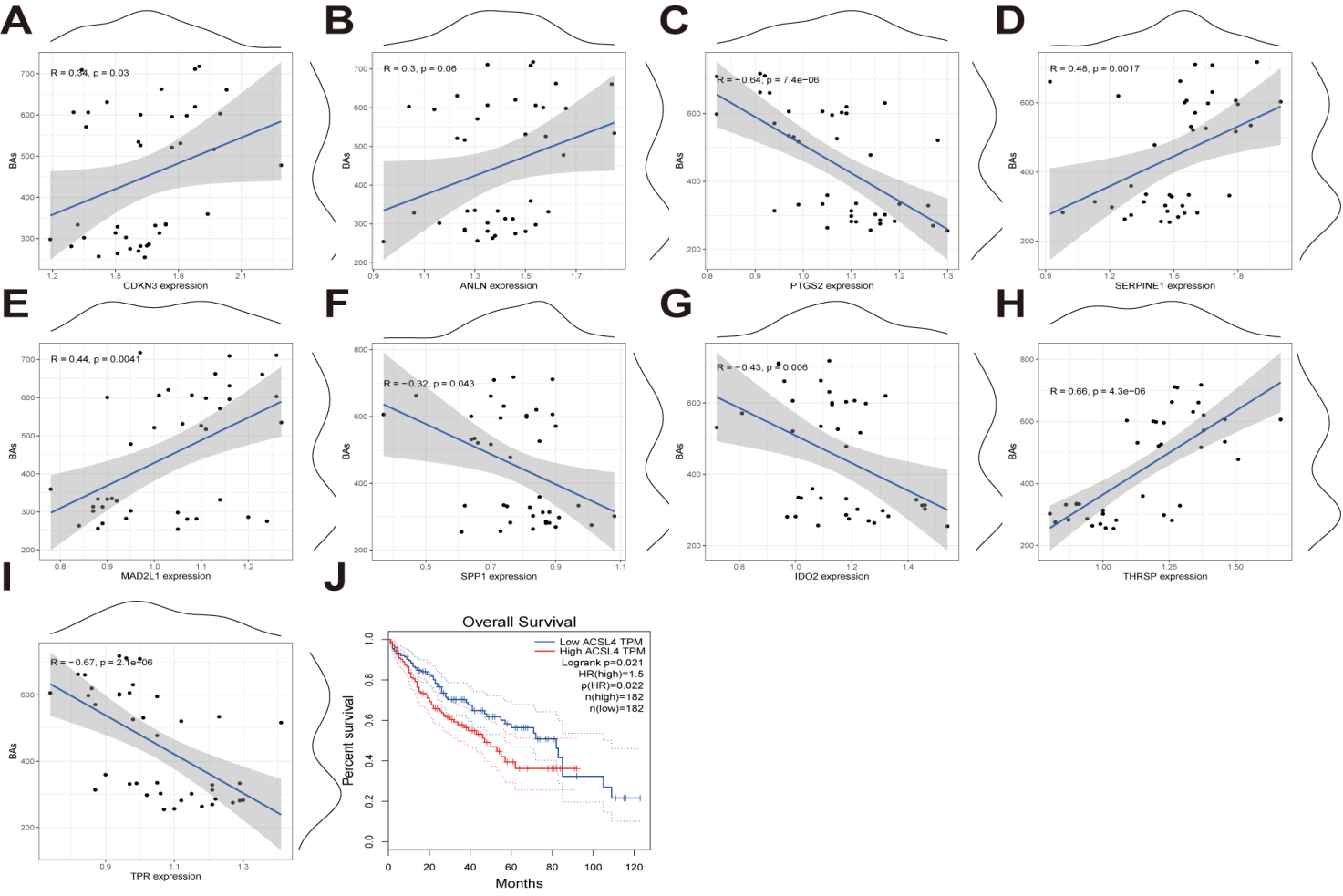


**Figure S1 Correlation Analysis and Survival Analysis Results.**

Note: A-I: Results of the correlation analysis between CDKN3, SERPINE1, PTGS2, ANLN, SPP1, TPR, MAD2L1, IDO2, and THRSP genes and bile acids, showing statistical differences with p<0.05. R represents the correlation coefficient, where R>0 indicates a positive correlation, and R<0 indicates a negative correlation. The closer the value is to 0, the weaker the correlation. J: According to the Kaplan-Meier survival curve, there is a significant difference in overall survival between the high ACSL4 expression group (red curve) and the low ACSL4 expression group (blue curve) (Logrank p-value <0.05). Patients with high ACSL4 expression exhibit significantly lower survival rates compared to those with low ACSL4 expression, indicating poor prognosis for patients with high ACSL4 expression.

**
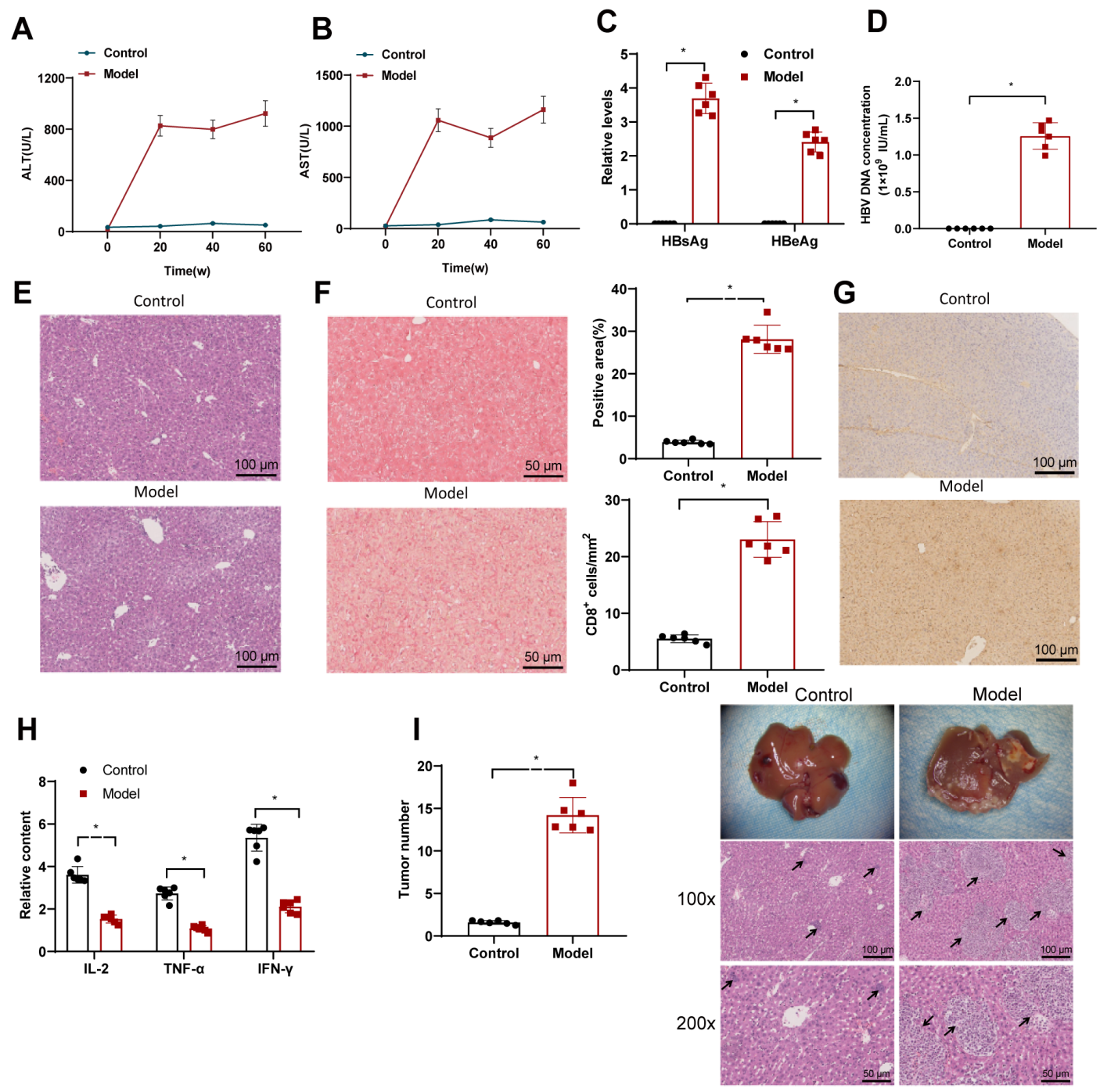
**

**Figure S2 Construction of HBs-HepR mouse model.**

Note: (A-B) Levels of ALT and AST in mouse serum measured by ELISA. (C) Levels of HBsAg and HBeAg in mouse serum measured by ELISA, n=6. Comparative analysis: Control vs. Experimental group, p<0.05. (D) Levels of HBV DNA in mouse serum measured by ELISA, n=6. Comparative analysis: Control vs. Experimental group, p<0.05. (E) Histopathological changes in mouse liver observed by H&E staining, scale bar: 50μm. (F) Fibrosis analysis of collagen deposition in mouse liver using Sirius Red staining, scale bar: 50μm. Comparative analysis: Control vs. Experimental group, p<0.05, n=6. (G) Detection of CD8+ cells by immunohistochemistry, scale bar: 50μm. Comparative analysis: Control vs. Experimental group, p<0.05, n=6. (H) Expression levels of IL-2, TNF-α, and IFN-γ in mouse liver tissue measured by ELISA, Comparative analysis: Control vs. Experimental group, p<0.05, n=6. (I) Morphology and nodule formation in mouse liver observed by H&E staining, black arrows indicate nodules, scale bar: 100 μm and 50μm. Comparative analysis: Control vs. Experimental group, p<0.05, n=6. Abbreviations: ALT: Alanine Aminotransferase; AST: Aspartate Aminotransferase; HBs-HepR: spontaneous HBV-related HCC mouse model.

**
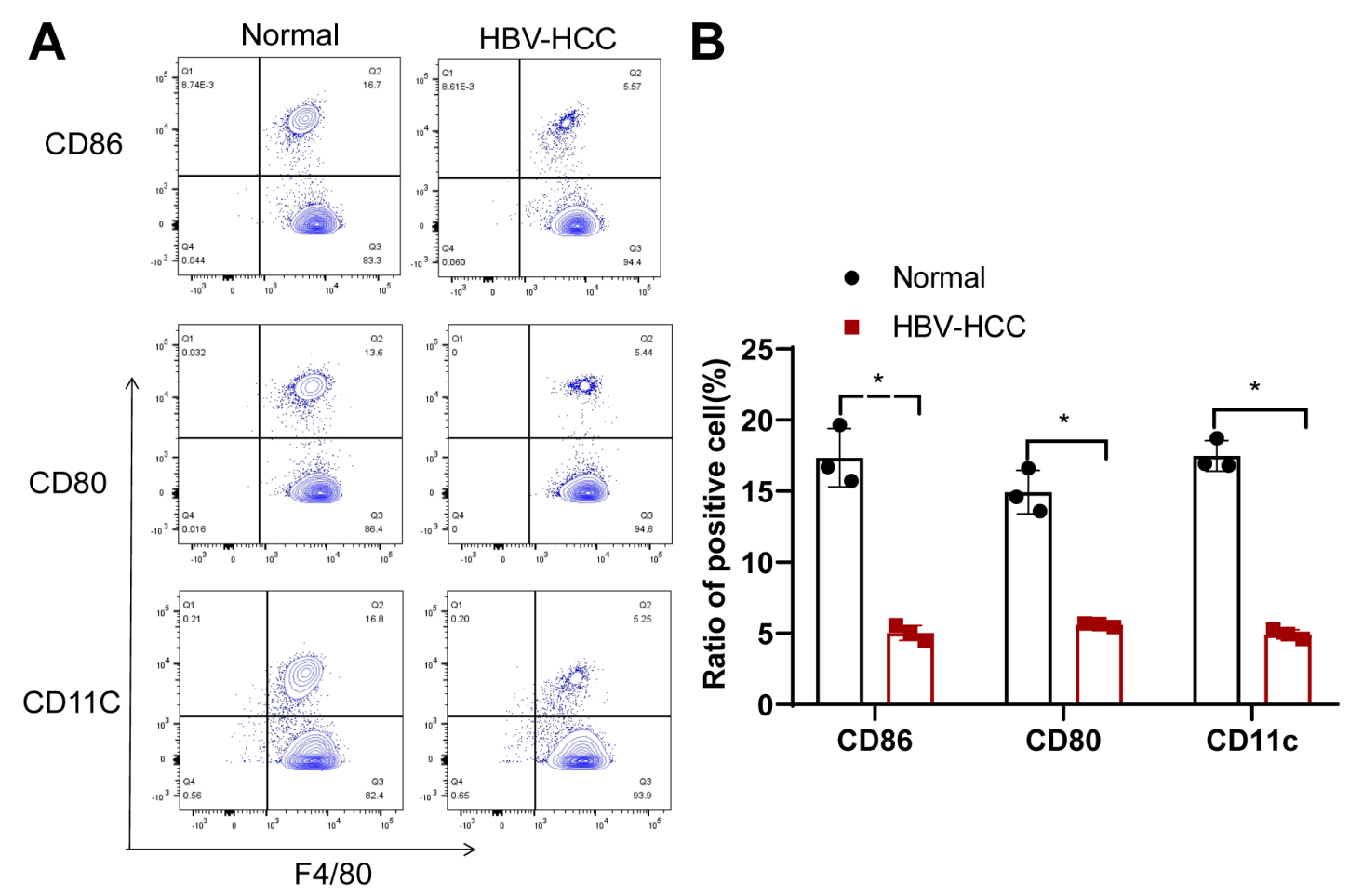
**

**Figure S3 The expression levels of polarization markers CD86, CD80, and CD11c in M1 macrophages detected by flow cytometry.**

Note: The difference between the two groups is statistically significant (p < 0.05).

**
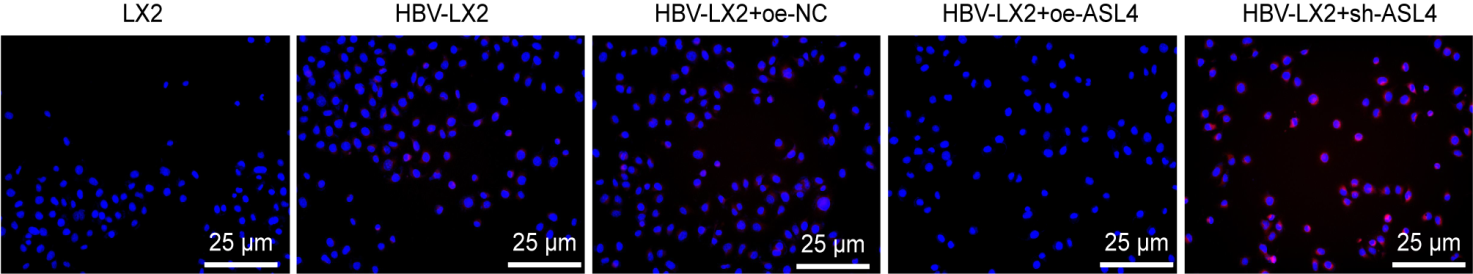
**

**Figure S4 Immunofluorescence detection of FerroOrange staining in HBV-LX-2 cells.**

Note: Scale bars, 25 μm.

**Table S1. ACSL4 knockdown sequence**

| **Name** | **Knockdown sequence** (5’-3’) |
| --- | --- |
| sh-ACSL4-1 | CCAGTGTTGAACTTCTGGAAA |
| sh-ACSL4-2 | CTTCCTCTTAAGGCCGGGAC |
| sh-FXR | TGGGATGTGCAGTGATGGAC |
| sh-NC | CCTAAGGTTAAGTCGCCCTCG |

**Table S2. RT-qPCRPrimer sequence**

| **Gene** | **Primer sequence (5’-3’)** |
| --- | --- |
| ACSL4 (human) | F：GGCACGCGGTTCCTTTTT |
|  | R： AGCCGACAATAAAGTACGCAA |
| CDKN3 (human) | F：AGCCGCCCAGTTCAATACAA |
|  | R：CTGTATTGCCCCGGATCCTC |
| SERPINE1 (human) | F：GCAAGGCACCTCTGAGAACT |
|  | R：GGGTGAGAAAACCACGTTGC |
| PTGS2 (human) | F：AGTCCCTGAGCATCTACGGT |
|  | R：CTCCACCAAAAGTGCTTGGC |
| ANLN (human) | F：TGTCTTCGTGGCCGATTTGA |
|  | R：ACTGTTTGTGCCAATGGTGC |
| SPP1 (human) | F：ATCTCCTAGCCCCACAGACC |
|  | R：GTGGGTTTCAGCACTCTGGT |
| TPR (human) | F：GCGACGTCTCCTAACCTGTG |
|  | R：GTTTTCCACCTTTTCTGAGGGT |
| MAD2L1 (human) | F：GGGCATGCAAATTGTGTCGT |
|  | R：ATGCTTGTTGGGGAGTCAGG |
| IDO2 (human) | F：AACCGAAGGATGGAACCTGG |
|  | R：TTCACATTCGGTCTGTGGGG |
| THRSP (human) | F：AACCATGCAGGTGCTAACCA |
|  | R：CCATGGTCCACATCCACACA |
| FXR (human) | F：GCAAAGAGATGGGAATGTTGGC |
|  | R：AGACCCCTCCCCTGTAATCC |
| GAPDH (human) | F：AATGGGCAGCCGTTAGGAAA |
|  | R：GCGCCCAATACGACCAAATC |
| ACSL4 (mouse) | F：GCTGAGAACTAAAGCCAGCA |
|  | R：CGTAGGATTCCACCAGCGTT |
| GAPDH(mouse) | F：GCCTCCTCCAATTCAACCCT |
|  | R：CTCGTGGTTCACACCCATCA |

Note: F, forward；R, reverse。

**Table S3. Western blot Antibody information**

| Protein name | Manufacturer | Article number | dilution ratio | Purchase Link |
| --- | --- | --- | --- | --- |
| ACSL4 | thermofisher | MA5-31543 | 1:1000 | https://www.thermofisher.cn/ |
| CDKN3 | thermofisher | PA5-106908 | 1:2000 | https://www.thermofisher.cn/ |
| PTGS2 | thermofisher | 35-8200 | 1 µg/mL | https://www.thermofisher.cn/ |
| SERPINE1 | Solarbio | K112064P | 1:1000 | https://www.solarbio.com/ |
| ANLN | thermofisher | PA5-28645 | 1:1000 | https://www.thermofisher.cn/ |
| SPP1 | Solarbio | K200110M | 1:1000 | https://www.solarbio.com/ |
| TPR | thermofisher | PA5-95533 | 0.2µg/mL | https://www.thermofisher.cn/ |
| MAD2L1 | Solarbio | K109473P | 1:1000 | https://www.solarbio.com/ |
| IDO2 | thermofisher | 703150 | 1:250 | https://www.thermofisher.cn/ |
| THRSP | thermofisher | PA5-77177 | 1:1000 | https://www.thermofisher.cn/ |
| FXR | thermofisher | 417200 | 1 µg/mL | https://www.thermofisher.cn/ |
| GAPDH | thermofisher | MA1-16757 | 1:1000 | https://www.thermofisher.cn/ |
